# The Sirt1 Activators SRT2183 and SRT3025 Inhibit RANKL-Induced Osteoclastogenesis in Bone Marrow-Derived Macrophages and Down-Regulate Sirt3 in *Sirt1* Null Cells

**DOI:** 10.1371/journal.pone.0134391

**Published:** 2015-07-30

**Authors:** Irina Gurt, Hanna Artsi, Einav Cohen-Kfir, Gilad Hamdani, Gal Ben-Shalom, Ben Feinstein, Madi El-Haj, Rivka Dresner-Pollak

**Affiliations:** 1 Endocrinology and Metabolism Service, Department of Medicine, Hadassah-Hebrew University Medical Center, Jerusalem, Israel; 2 The Einstein Institute of Mathematics; The Hebrew University of Jerusalem, Jerusalem, Israel; 3 Department of Orthopedics, Hadassah-Hebrew University Medical Center, Jerusalem, Israel; China Medical University, TAIWAN

## Abstract

Increased osteoclast-mediated bone resorption is characteristic of osteoporosis, malignant bone disease and inflammatory arthritis. Targeted deletion of Sirtuin1 (Sirt1), a key player in aging and metabolism, in osteoclasts results in increased osteoclast-mediated bone resorption *in vivo*, making it a potential novel therapeutic target to block bone resorption. Sirt1 activating compounds (STACs) were generated and were investigated in animal disease models and in humans however their mechanism of action was a source of controversy. We studied the effect of SRT2183 and SRT3025 on osteoclastogenesis in bone-marrow derived macrophages (BMMs) *in vitro*, and discovered that these STACs inhibit RANKL-induced osteoclast differentiation, fusion and resorptive capacity without affecting osteoclast survival. SRT2183 and SRT3025 activated AMPK, increased Sirt1 expression and decreased RelA/p65 lysine310 acetylation, critical for NF-κB activation, and an established Sirt1 target. However, inhibition of osteoclastogenesis by these STACs was also observed in BMMs derived from *sirt1* knock out (*sirt1^-/-^)* mice lacking the Sirt1 protein, in which neither AMPK nor RelA/p65 lysine 310 acetylation was affected, confirming that these effects require Sirt1, but suggesting that Sirt1 is not essential for inhibition of osteoclastogenesis by these STACs under these conditions. In *sirt1* null osteoclasts treated with SRT2183 or SRT3025 Sirt3 was found to be down-regulated. Our findings suggest that SRT2183 and SRT3025 activate Sirt1 and inhibit RANKL-induced osteoclastogenesis *in vitro* however under conditions of Sirt1 deficiency can affect Sirt3. As aging is associated with reduced Sirt1 level and activity, the influence of STACs on Sirt3 needs to be investigated *in vivo* in animal and human disease models of aging and osteoporosis.

## Introduction

Increased bone resorption by osteoclasts is characteristic of osteoporosis, inflammatory arthritis, hyperparathyroidism, malignant bone disease and other metabolic bone diseases. Currently available therapies to suppress osteoclast-mediated bone resorption include the bisphosphonates which induce osteoclast apoptosis and may result in suppression of bone formation and anti receptor activator of nuclear factor-κB ligand (RANKL)-antibody. These therapeutic agents are precluded from long term use due to side effects. Sirtuin 1 (Sirt1), a nicotinamide adenine dinucleotide (NAD^+^)-dependent lysine deacetylase, a key player in aging, inflammation and metabolism [1] regulates bone mass, and its targeted deficiency in osteoclasts results in increased bone resorption [2–6]. Enhancing Sirt1 activity is a plausible novel approach to inhibit bone resorption while concurrently ameliorating other age-related pathologies.

Resveratrol, the first Sirt1 activator to be studied, inhibits osteoclast generation and function [7], but this effect may be mediated via its cellular targets beyond Sirt1 such as estrogen receptor alpha, a key regulator of osteoclast generation [8] and influenced by resveratrol [9]. Synthetic Sirtuin 1 activating compounds (STACs), structurally different than resveratrol with a higher potency and bioavailability were generated, however their mechanism of action was a source of ongoing debate [10–13]. The controversy seemed to have been resolved by a study showing an allosteric activation of Sirt1 by STACs requiring hydrophobic motifs in the substrates and glutamic acid at position 230 of the Sirt1 N-terminal domain [14]. Different STACS were extensively tested in a wide spectrum of disease models in animals and over the past few years in humans in patients with type 2 diabetes mellitus and inflammatory conditions [15–17]

Osteoclast-mediated bone resorption is a high energy demanding process [18] and sensors of cellular energy are likely to play a role in it. In this study we investigated the effects of second and third generations STACs [19] on osteoclast generation and function *in vitro*, and discovered that SRT2183 and SRT3025 inhibit RANKL-induced osteoclastogenesis in bone marrow-derived macrophages (BMMs) by activating AMPK and deacetylating RelA/p65 lysine 310, critical for activation of the NF-κB signaling pathway. However, inhibition of osteoclastogenesis was also observed in SRT2183 and SRT3025-treated bone marrow macrophages derived from *sirt1* knock-out mice in which neither AMPK nor RelA/p65 lysine 310 acetylation was affected but Sirt3 was down-regulated. Our findings suggest that these STACs inhibit osteoclastogenesis and can down-regulate Sirt3 under conditions of Sirt1 deficiency.

## Methods

### Animals

8-week-old female 129/Sv mice were used for this study. Inbred 129/Sv *Sirt1* ^*+/Δ*^ mice [20] were a generous gift (see Acknowledgments), and were used for generating *Sirt1* ^*Δ/Δ*^ (*Sirt1* ^*-/-*^) mice and the littermates of the parental WT strain. Genotyping of mice was performed at 4 weeks of age using ear genomic DNA as templates. All mice were maintained under specific pathogen-free conditions. Mice were housed in a constant temperature room with a 12-hour dark/12-hour light circle and were allowed free access to standard chow and water. Mice were sacrificed by isoflurane inhalation (Minrad INC, USA). All experiments were performed with the approval of the Animal Study Committee of the Hebrew University-Hadassah Medical School (MD-12-13154-3).

### *In vitro* assays of osteoclast differentiation

Bone marrow-derived macrophages (BMMs) from femurs and tibias were collected, plated, and non-adherent cells were re-plated 24-hrs later in a 96-well plate at a concentration of 20,000 cells/well unless otherwise specified. The cells were cultured for 3 days in 5% CMG14–12 culture supernatant as a source of macrophage-colony stimulating factor (M-CSF) [21] in minimum essential medium α (α–MEM) containing 15% FBS. The plated cells were then induced to differentiation with 10% M-CSF and 10 ng/ml RANKL (PeproTech, Rocky Hill, New Jersey) for 4 days with a medium change every 3 days. Cells were TRAP-stained using a commercial kit (Sigma-Aldrich product 387-A, St. Louis, MO). Four non-overlapping images representing 80% of the area of each well were photographed with the Nikon DS Fi1 camera attached to Nikon Eclipse 80i microscope. Octeoclasts, defined as TRAP-positive multi-nucleated (≥3 nuclei) cells, were manually counted.

### Compounds

**Fig 1:**
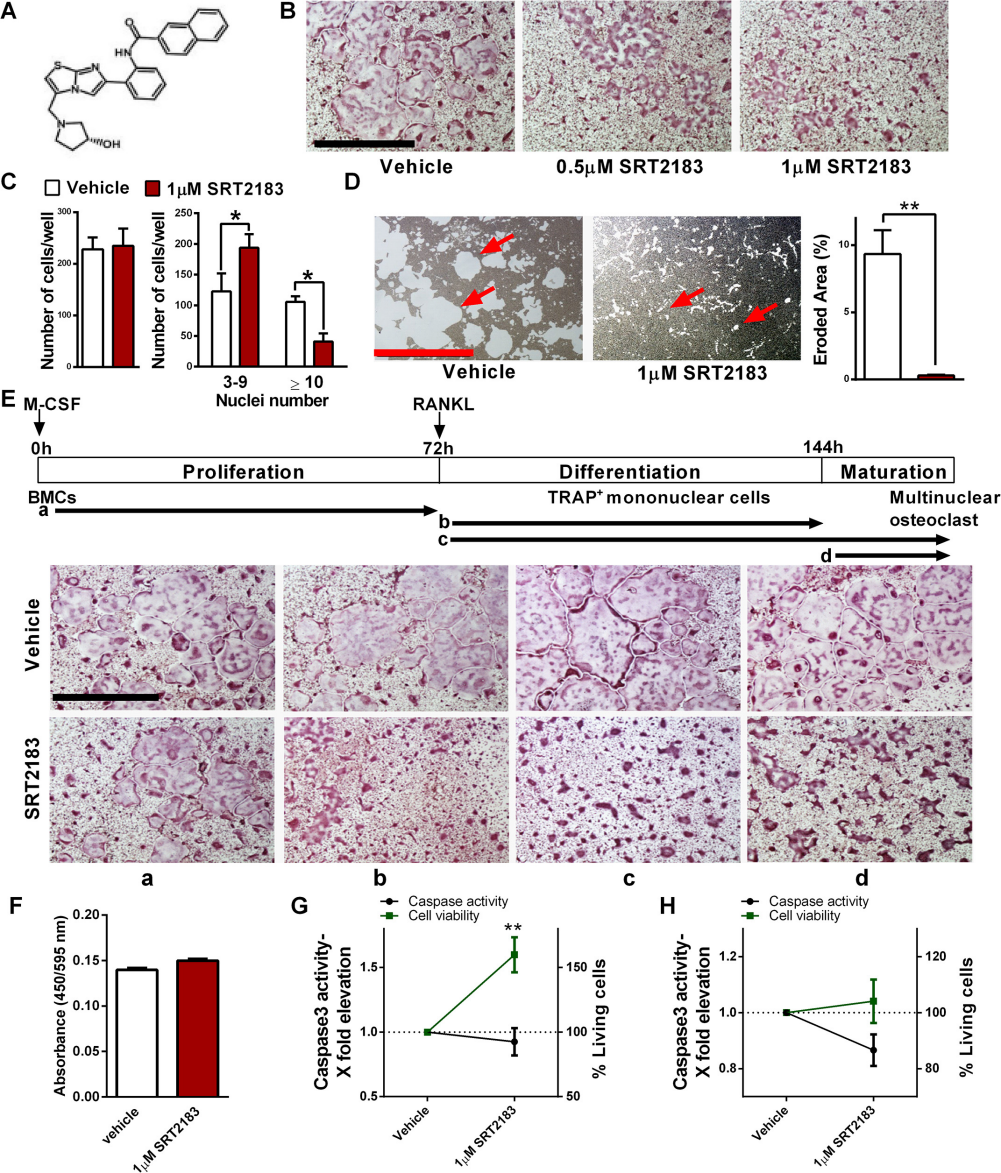
SRT2183 inhibits RANKL-induced osteoclastogenesis in bone marrow-derived macrophages (BMMs). (A) Chemical structure of SRT2183. (B-C) TRAP staining of SRT2183 or vehicle-treated BMMs inducted to osteoclastogenesis 4 days post RANKL stimulation (B). Total number of osteoclasts (left panel) and number of multinuclear cells (right) are shown (C). (D) The effect of SRT2183 on pit formation by RANKL-induced BMMs. A pit formation assay (left panel) and eroded area (right) are shown. (E) Time course of the effect of SRT2183 or vehicle (DMSO) administration on osteoclast differentiation. SRT2183 or a vehicle was added in the proliferation (a), differentiation (b, c) or maturation (c, d) phase. Arrows indicate periods of treatments with SRT2183. TRAP staining is shown. (F) The effect of SRT2183 on cell proliferation during the proliferation phase. SRT2183 or a vehicle were co-administrated with M-CSF for 72 hours on day of plating. (G-H) The effect of SRT2183 on cell viability and apoptosis during the proliferation phase (G) and the differentiation and maturation phase (H). SRT2183 or vehicle were co-administrated with M-CSF for 72 hours on day of plating (G) or with RANKL 3 days post plating (H). The graphs illustrate fold change in Caspase 3 activity and the percent change in living cells with time. Data are Mean ±SEM (n = 3 independent experiments), analyzed by 2 way ANOVA with nuclei number and treatment as the independent variables followed by Sidak's post-hoc correction (C), paired Student's *t*-test (D), one-sample Student's *t*-test (F-H), *P<0.05; **P<0.01 compared to vehicle-treated BMMs. Magnification X40; Scale bar 1mm.

SRT2183 (Fig 1A) and SRT3025, kindly provided by Sirtris-GSK (see Acknowledgments), were dissolved in DMSO and were co-administrated with RANKL, unless otherwise specified. The compounds or the vehicle (0.01% DMSO) were added upon each medium exchange. All experiments were conducted with SRT2183 and some key experiments were repeated with SRT3025. Initial dose-response experiments with 0.5, 1, 2μM SRT2183 and 1, 2, 5μM SRT3025 were conducted based on the manufacturer recommendation, and TRAP staining suggested that the 2μM and 5μM concentrations are toxic for SRT2183 and SRT3025, respectively. All experiments were therefore conducted with 1μM SRT2183 and 2μM SRT3025. For the time course studies SRT2183, SRT3025 or a vehicle was added in the proliferation phase (co-administrated with M-CSF on day of plating and removed 3 days post plating), differentiation phase (co-administrated with RANKL on day 4 and removed on day 7), maturation phase only (day 7 post plating for 24 hrs) or differentiation and maturation phases (co-administrated with RANKL on day 4).

### Pit formation assay

BMMs were harvested, plated and 24-hrs later non-adherent cells were re-plated on Osteo Assay Plate with an inorganic crystalline calcium phosphate coating (Corning, NY-cat no CI-3988) [22] at a density of 20,000 cells/well in α–MEM/15%FBS/5% M-CSF. On day 4 cells were induced to differentiation with 10% M-CSF/20ng/ml RANKL in the presence of SRT2183, SRT3025 or a vehicle for 7 days. Higher doses of RANKL compared to the differentiation assays were used for this experiment as cells were maintained for a longer period of time in culture. On day 11 adherent osteoclasts were removed using sodium hypochlorite solution (Sigma-Aldrich, St. Louis, MO) and the resorption area was determined by a Nikon ecplise 80i microscope coupled color camera Nikon DS (Digital sight)-Fi1 and IMAGE-PRO EXPRESS 4.0 software (Media Cybernetics, Silver Spring, MD). Resorption area was quantified by MATLAB Image Processing Toolbox (MATLAB R2013a) and is presented as percentage of well area.

### Gene expression analyses

RNA was extracted from osteoclasts using peqGOLD TriFast (PeqLab, Erlangen, Germany) at the indicated time points, reverse transcribed into cDNA and analyzed with SYBR Green-based quantitative Real-Time PCR in triplicates. mRNA expression level was normalized to *Gapdh*, *β-actin* or RNA polymerase II *Polr2a*. *Gapdh* is commonly used as a reference gene in osteoclast studies and indeed was stable in our experiments. To confirm that it does not affect the results when analyzing genes involved in energy metabolism we also used the mentioned above genes.

Antibodies to the following proteins were used for western blotting: NFATc1 (sc-7294, Santa Cruz), DC-STAMP (MABF39, Millipore), Sirt1 (07–131, Millipore), Sirt3 (#5490, Cell signaling), acetylated Sod2 (acetyl-superoxide dismutase K68, ab137037, Abcam), Sod2 (ab16956, Abcam), phosphorylated AMPKα (AMP-activated protein kinase, #2535, Cell Signaling), AMPKα (#2793, Cell Signaling), phosphorylated ACC (Acetyl CoA Carboxylase, #3661, Cell Signaling), ACC (#3662, Cell Signaling), acetylated p65 (Acetyl-NF-κB p65(Lys310) #3045, Cell signaling), p65 (#3987, Cell signaling), IκBα (#4814, Cell signaling), HSP90 (610419, BD Transduction laboratories) and GAPDH (ab8245, Abcam, UK) were used as reference proteins.

### Proliferation assay

BMMs were harvested, plated and 24-hrs later non-adherent cells were re-plated at a density of 20,000 cells/well in α–MEM/15%FBS/5% M-CSF. For determining cell proliferation, cells were treated with SRT2183 or a vehicle for 72 hours post plating and BrdU reagent (Abcam, UK) was added 48hrs post SRT2183 or vehicle administration according to the manufacturer's instructions to determine cell proliferation 3 days post plating.

### Cell viability assay

BMMs were harvested, plated and 24-hrs later non-adherent cells were re-plated at a density of 20,000 cells/well in α–MEM/15%FBS/5% M-CSF. For determining cell viability during the proliferation phase, cells were treated with SRT2183 or a vehicle for 72 hours post plating and cellTiter-Blue reagent (Promega, Madison, Wis) was added according to the manufacturer's instructions to determine cell survival on day 4. For determining cell viability during the differentiation and maturation phases, cells were induced to differentiation on day 4 with 10% M-CSF/10ng/ml RANKL in the presence of SRT2183 or a vehicle, and cellTiter-Blue reagent was added to determine cell survival on day 8 post plating.

### Apoptosis assay by Caspase 3 activity

BMMs were harvested, plated and 24-hrs later non-adherent cells were re-plated at a density of 20,000 cells/well in α–MEM/15%FBS/5% M-CSF. For apoptosis determination during the proliferation phase, cells were treated with SRT2183 or a vehicle for 72 hours and Caspase 3 activity within the cells was assessed by using the Apo-ONE Homogeneous Caspase 3/7 Assay Kit (Promega) on day 4.

For apoptosis determination during the differentiation and maturation phases, cells were induced to differentiation on day 4 with 10% M-CSF/10ng/ml RANKL in the presence of SRT2183 or a vehicle. Caspase 3 activity within the cells was assessed with the same kit on day 8. Experiments were carried out 3 times in parallel with cell viability assays.

### Statistical analysis

Results are presented as Mean ± SEM. Data was analyzed by 2- way ANOVA, one or two sample Student's *t*-test as appropriate to compare treated versus untreated cells using graphPad Prism version 6 (software San Diego CA). Each experiment was repeated at least 3 times. *P*-values less than 0.05 were considered significant.

## Results

### SRT2183 inhibits RANKL-induced osteoclast generation and resorptive capacity in bone marrow macrophages

The generation of multi-nucleated osteoclasts as determined by TRAP staining was significantly hampered when BMMs were induced to osteoclastogenesis in the presence of SRT2183 in a dose-dependent manner (Fig 1B). Total osteoclast number was not affected by SRT2183 administration, but the generation of large osteoclasts with a high nuclei number was markedly decreased (Fig 1C). Importantly, SRT2183 dramatically inhibited osteoclast resorptive capacity as indicated by a marked reduction in the area eroded by treated cells (Fig 1D).

**Fig 2:**
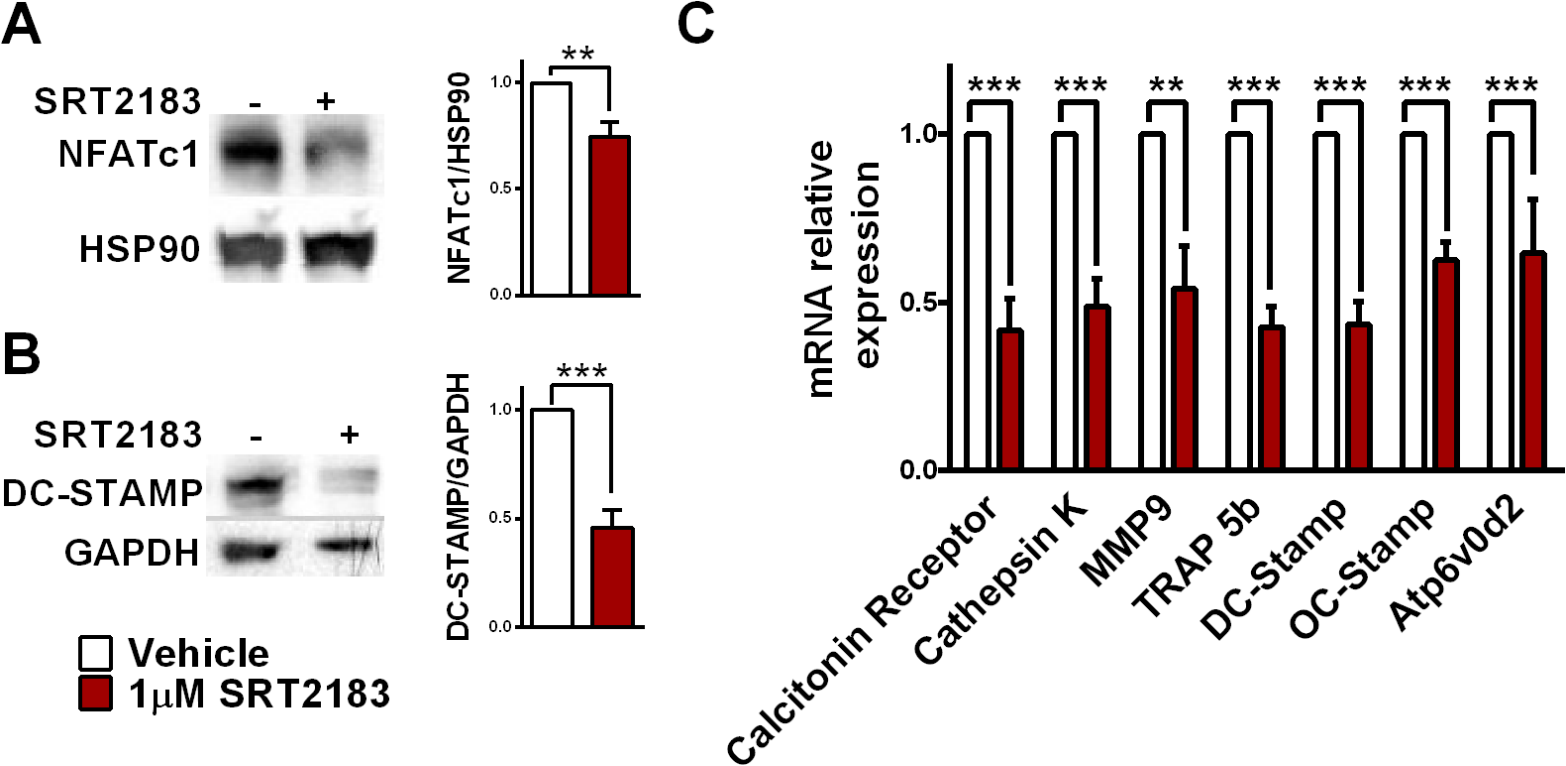
SRT2183 inhibits RANKL-induced NFATc1 activation in bone marrow-derived macrophages (BMMs). (A) The effect of SRT2183 on NFATc1 protein level. Western blot analysis of NFATc1 and HSP90 in SRT2183- and vehicle-treated osteoclasts 4 days post RANKL stimulation. NFATc1- nuclear factor of activated T-cell cytoplasmic 1. (B) The effect of SRT2183 on DC-STAMP protein level. Western blot analysis of DC-STAMP and GAPDH in SRT2183- and vehicle-treated osteoclasts 4 days post RANKL stimulation. DC-STAMP- dendritic cell-specific transmembrane protein. (C) The effect of SRT2183 on mRNA expression of osteoclast markers and fusion-related genes. SRT2183 or vehicle were co-administrated with RANKL. Gene expression analysis by quantitative Real-Time PCR 4 days post RANKL stimulation is shown. Results are relative to *GAPDH*. Data are Mean ±SEM (n = 3 independent experiments), analyzed by one-sample Student's *t*-test, *P<0.05; **P<0.01; ***P<0.001 compared to vehicle-treated BMMs.

Time course experiments revealed that SRT2183 treatment at the proliferation phase did not affect osteoclast generation, however osteoclast differentiation and maturation were markedly reduced when SRT2183 was administered at the differentiation stages (Fig 1E a-d). The proliferation of osteoclast precursors was not altered by SRT2183 treatment (Fig 1F). To understand if SRT2183 affects cell survival, viability and apoptosis studies were conducted at the proliferation and differentiation phases. The administration of SRT2183 at the proliferation and differentiation phases did not decrease cell viability or increased apoptosis (Fig 1G and 1H). Accordingly, total protein was unchanged in SRT2183 versus vehicle-treated cells (S1 Fig). These results suggest that SRT2183 inhibits osteoclast differentiation and function but not precursors’ proliferation or cell survival. Consistently, nuclear factor of activated T-cell cytoplasmic 1 (NFATc1), a master transcription factor in osteoclast differentiation [23] as well as its downstream target, dendritic cell-specific transmembrane protein (DC-STAMP), necessary for osteoclast multi-nucleation [24] were reduced in SRT2183-treated cells (Fig 2A and 2B). Similarly, mRNA expression of osteoclast markers and specifically key osteoclast fusion-related genes, Tm7sf4 (encoding for DC-STAMP) and the gene encoding for osteoclast stimulatory trans-membrane protein (OC-STAMP) were significantly decreased in SRT2183-treated osteoclasts (Fig 2C).

### SRT2183 activates AMPK in osteoclasts

To gain insight into the mechanism by which SRT2183 inhibits RANKL-induced osteoclast differentiation and function major signaling pathways downstream of RANK were screened. Early phosphorylation of: c-jun N-terminal kinase (JNK), mitogen-activated protein kinase 14 (p38), ERK and p65 were not affected by SRT2183 administration (S2 Fig).

**Fig 3:**
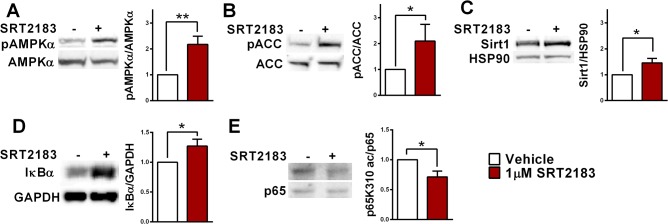
SRT2183 activates AMPK and deacetylates RelA/p65 K310 in RANKL-induced BMMs. (A) The effect of SRT2183 on AMPKα phosphorylation (Thr172). Western blot analysis of pAMPKα and AMPKα in SRT2183- and vehicle-treated osteoclasts 4 days post RANKL stimulation. p- phosphorylated; AMPKα- AMP-activated protein kinase alpha. (B) The effect of SRT2183 on ACC phosphorylation. Western blot analysis of pACC and ACC in SRT2183- and vehicle-treated osteoclasts 4 days post RANKL stimulation. p- phosphorylated; ACC-Acetyl CoA Carboxylase. (C) The effect of SRT2183 on Sirt1 protein level in RANKL-stimulated osteoclasts. Western blot analysis of Sirt1 and HSP90 in SRT2183- and vehicle-treated osteoclasts 4 days post RANKL stimulation. (D) The effect of SRT2183 on IκBα protein level. Western blot analysis of IκBα and GAPDH in SRT2183- and vehicle-treated BMMs 24 hours post RANKL stimulation. (E) The effect of SRT2183 on p65 acetylation (Lys310). Western blot analysis of p65K310 ac and p65 in SRT2183- and vehicle-treated osteoclasts 4 days post RANKL stimulation. Data are Mean ±SEM (n = 3 independent experiments), analyzed by one-sample Student's *t*-test; *P<0.05 versus vehicle-treated BMMs.

Previous work has shown that Sirt1 is closely coupled to AMP-activated protein kinase (AMPK) activity in a mutually enforcing mechanism [25]. Moreover, AMPK regulates osteoclast differentiation and function, and AMPKα1 deficiency in mice causes enhanced osteoclast differentiation and fusion [26]. We therefore investigated AMPK activation and discovered increased phosphorylation of AMPKα and its target acetyl CoA carboxylase (ACC) in SRT2183-treated cells, indicating AMPK stimulation (Fig 3A and 3B). Of note, increased Sirt1 level in SRT2183-treated cells was also observed (Fig 3C), and can result from AMPK activation, as AMPK was shown to positively regulate Sirt1 level [27].

### SRT2183 regulates factors of the NF-κB signaling pathway in osteoclasts

Both Sirt1 and AMPK were shown to inhibit NF-κB signaling, a key pathway in RANKL-induced osteoclastogenesis [28]. Sirt1 represses NF-κB transcriptional activity by deacetylating RelA/p65 at lysine 310, critical for NF-κB activation [29]. AMPK was shown to inactivate the NF-κB pathway via inhibition of IκB kinase and IκBα degradation. IκBα is an inhibitory subunit complexed to NF-κB /Rel proteins in the cytoplasm, preventing the release and movement of NF-κB into the nucleus [30]. Along these lines, decreased IκBα was reported in osteoclasts derived from *AMPKα1* ^*-/-*^ mice [26]. Indeed, IκBα was markedly increased and RelA/p65 K310 acetylation was significantly decreased in SRT2183-treated osteoclasts (Fig 3D and 3E). These results suggest that SRT2183 activates Sirt1 and AMPK in bone marrow-derived osteoclasts leading to inhibition of RANKL-induced NF-κB activation and NFATc1 expression.

### SRT2183 inhibits osteoclastogenesis in sirt1^-/-^-derived bone marrow macrophages

**Fig 4:**
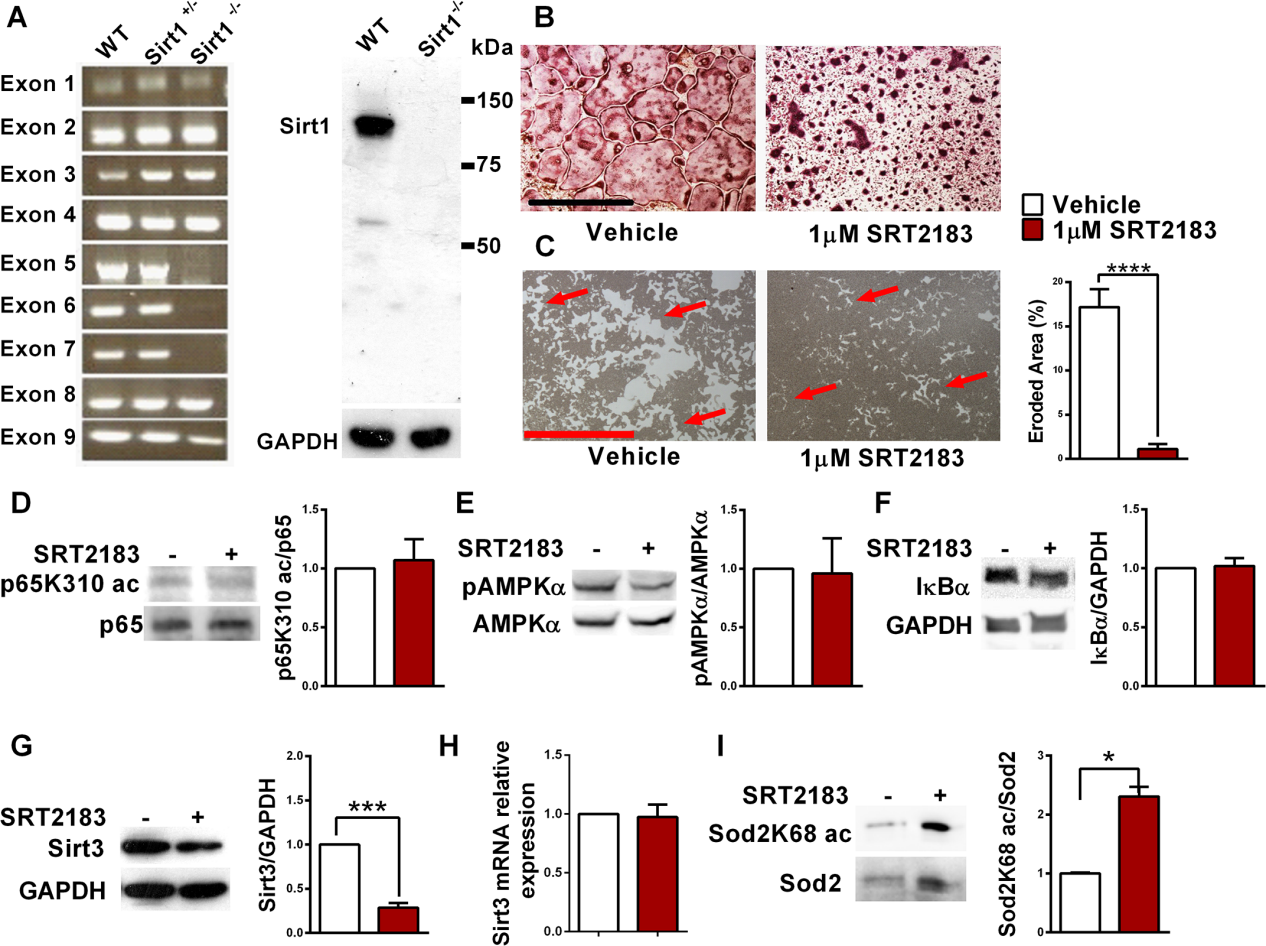
SRT2183 inhibits RANKL-induced osteoclastogenesis and pit formation in s*irt1* ^*-/-*^ BMMs. (A) Sirt1 expression in WT- and in *Sirt1* ^*-/-*^-derived osteoclasts. PCR amplification of exons 1–9 of the s*irt1* gene (left panel) and Western blot analysis with Sirt1 antibody (right panel) demonstrates complete loss of Sirt1 protein in osteoclasts obtained from *Sirt1* ^*Δ/Δ*^ (*Sirt1* ^*-/-*^) mice. (B) The effect of SRT2183 on osteoclast differentiation in *Sirt1* ^*-/-*^-derived BMMs. BMMs were inducted to osteoclastogenesis with RANKL in the presence or absence of SRT2183. TRAP staining performed 4 days post induction. (C) The effect of SRT2183 on pit formation in *Sirt1* ^*-/-*^-derived BMMs stimulated with RANKL. An eroded area (left panel) and pit formation assay (right) are shown. (D) The effect of SRT2183 on p65 acetylation (Lys310). Western blot analysis of p65K310 ac and p65 in SRT2183- and vehicle-treated osteoclasts 4 days post RANKL stimulation. (E) The effect of SRT2183 on AMPKα phosphorylation (Thr172). Western blot analysis of pAMPKα and AMPKα in SRT2183- and vehicle-treated osteoclasts 4 days post RANKL stimulation. (F) The effect of SRT2183 on IκBα protein level. Western blot analysis of IκBα and GAPDH in SRT2183- and vehicle-treated BMMs 24 hours post RANKL stimulation. (G-H) The effect of SRT2183 on Sirt3 protein (G) and gene expression (H). Western blot analysis of Sirt3 and GAPDH in SRT2183- and vehicle-treated osteoclasts 4 days post RANKL stimulation (G). Gene expression analysis by quantitative Real-Time PCR 4 days post RANKL stimulation is shown. Results are relative to *Polr2a* (H). (I) The effect of SRT2183 on superoxide dismutase 2 (Sod2) Lys68 acetylation. Western blot analysis of acetylated (ac) Sod2K68 and Sod2 in SRT2183- and vehicle-treated osteoclasts 4 days post RANKL stimulation. Data are Mean ± SEM (n = 3 independent experiments), analyzed by paired Student's *t*-test paired (C) or one-sample Student's *t*-test (H-I); ***P<0.001, ****P<0.0001, versus vehicle-treated BMMs. Magnification X40; scale bar 1mm.

To understand the role of Sirt1, the influence of SRT2183 on osteoclastogenesis was evaluated in bone marrow cells derived from *sirt1* ^-/-^ mice. *Sirt1* ^*Δ/Δ*^ (*Sirt1* ^*-/-*^) mice lacking Sirt1 protein were generated from inbred 129/Sv *Sirt1* ^*+/Δ*^ mice, whereas their littermates WT served as the controls. These KO mice are lacking *sirt1* exons 5–7 resulting in no sirt1 protein production (Fig 4A) and have a birth rate lower than 3% [20]. Strikingly, SRT2183 abolished the generation of large multi-nucleated osteoclasts and their resorptive capacity in *sirt1* ^*-/-*^ BMMs similar to the effect observed in WT-derived osteoclasts, indicating that Sirt1 is not essential for inhibition of osteoclast generation and function under these conditions (Fig 4B and 4C). As expected, RelA/p65 K310 acetylation was not changed in SRT2183-treated *sirt1* ^*-/-*^ osteoclasts (Fig 4D), as this is a direct Sirt1 target. Furthermore, AMPKα phosphorylation was not affected by SRT2183 administration in *sirt1* ^*-/-*^-derived osteoclasts (Fig 4E), suggesting that Sirt1 is upstream of and is required for AMPK activation by SRT2183 under these conditions. Consistently IκBα level was unchanged (Fig 4F).

### SRT2183 down-regulates Sirt3 in *sirt1* ^*-/-*^-derived bone marrow macrophages

To better understand the mechanism by which osteoclastogenesis is inhibited in SRT2183-treated *sirt1* ^*-/-*^ osteoclasts, we first asked if Sirtuins 2–7 protein level is changed in *sirt1* ^*-/-*^-derived bone marrow macrophages or osteoclasts. No change was detected (S3 Fig). Next, we asked if Sirt2-7 protein level is modulated by SRT2183 administration in WT or *sirt1* KO cells. Strikingly, while no change was observed in treated WT osteoclasts (S4 Fig), Sirt3 protein but not mRNA expression was significantly reduced in *sirt1* ^*-/-*^-treated osteoclasts (Fig 4G and 4H). Moreover, decreased Sirt3 activity was detected in SRT2183-treated *sirt1* null cells as indicated by increased acetylation of its target

manganese superoxide dismutase (MnSOD, Sod2) [31] (Fig 4I). Finally, we asked if inhibition of osteoclastogenesis occurs also with a more advanced STAC, such as SRT3025, a third generation STAC [19]. Similar effects of SRT3025 on inhibition of osteoclastogenesis were observed in both WT (S5 Fig) and in *sirt1* null cells (S6 Fig). Consistently, Sirt3 was reduced in *sirt1* null cells treated with SRT3025. Thus, both SRT2183 and SRT3025 inhibited RANKL-induced osteoclastogenesis independently of Sirt1 and down-regulated Sirt3 in *sirt1* null cells.

## Discussion

This study demonstrates that Sirt1 activating compounds (STACs), SRT2183 and SRT3025, inhibit RANKL-induced osteoclast differentiation, multi-nucleation and resorptive capacity in bone marrow derived-macrophages *in vitro* without hampering cell survival. SRT2183 and SRT3025 inhibited RANKL-induced osteoclast differentiation by promoting deacetylation of RelA/p65 at lysine 310, a well recognized direct Sirt1 target [29] critical for NF-κB activation [32], and by activating AMPK. RelA/p65 was previously shown to promote osteoclast differentiation by blocking a RANKL-induced apoptotic JNK pathway, leading to enhanced OC differentiation [33]. Moreover, mice lacking RelA/p65 in the hematopoietic compartment have a deficient osteoclastogenic response to RANKL [33]. RelA/p65 contains seven lysine acetylation sites of which lysine 310 acetylation is required for NF-κB transcription activation and was shown to be deacetylated by Sirt1 [34]. In agreement with our results, targeted *sirt1* deletion in osteoclasts leads to increased osteoclastogenesis and bone resorption accompanied by elevated osteoclast RelA/p65 lysine 310 acetylation [6]. Consistently, deacetylation of RelA/p65 K310 by STACs was previously described in U20S or HEK293 cells [35]. SRT2183 and SRT3025 activated AMPK via Sirt1 stimulation, as indicated by lack of effect on AMPK in *sirt1* null cells. An intimate interplay between Sirt1 and AMPK was previously shown in a number of cell types. Sirt1 deacetylases and activates serine-threonine liver kinase B1 (LKB1), the primary AMPK kinase and activator [36]. On the other hand, AMPK increases Sirt1 expression and function via increasing its co-factor NAD^+^ [27,37]. AMPK inhibits osteoclastogenesis by inhibiting the NF-κB pathway in part by preventing the degradation of IκBα a repressor of NF-κB which holds it quiescent in the cytoplasm [38]. The physiologic significance of this effect is illustrated by the phenotype in IκBα haplo-insufficient mice which display increased osteoclastogenesis [6].

Suppression of osteoclastogenesis by SRT2183 and SRT3025 occurred also in BMMs derived from *sirt1* ^*-/-*^ mice. These findings are in disagreement with some previously published studies which reported no effect of STACs in *sirt1* deficient cells [14,39]. However, other previously published studies reported Sirt1-independent effects of STACs [10,40]. The discrepancy may be explained at least in part by the fact that in those studies claiming no off target effects only *sirt1* exons 4–5, encoding for the enzyme catalytic domain, were deleted, allowing for STACs binding to inactive existing Sirt1 protein and precluding their binding to other targets, whereas in our study cells with complete deletion of *sirt1* were investigated. It is also plausible that sirt1 specificity of these compounds is cell-dependent and osteoclasts were never studied before.

We discovered Sirt3 to be a target of SRT2183 and SRT3025 in *sirt1* null cells resulting in its down-regulation, thus these STACs had an inhibitory action rather than being activators under these conditions. Supporting our findings, SRT1720 was previously shown to inhibit mouse and human Sirt3 by partially occupying the acetyl-lysine binding site, thus competing with the peptide substrate [41,42]. Furthermore, resveratrol, the first described Sirt1 activator, was also shown to inhibit Sirt3 [43]. The mechanism by which reduced Sirt3 in *sirt1* ^*-/-*^ osteoclasts leads to inhibition of osteoclastogenesis is not completely understood, however an increase in the acetylated inactive form of MnSod2 was found in SRT2183- treated *sirt1* null cells,. The role of Sod2 in osteoclasts is unknown. Sod2was identified as a susceptibility gene for osteoporosis in humans. SNPs in the Sod2 gene were to be translated into changes in mRNA transcription and protein expression, and Sod2 protein expression is inversely associated with BMD in the Chinese population, suggesting that low Sod2 may be bone protective [44,45].

In summary, this study demonstrates that the STACs SRT2183 and SRT3025 inhibit osteoclast generation and function *in vitro*. These compounds did not cause osteoclast apoptosis and therefore are unlikely to impair the coupling between osteoclasts and osteoblasts. Whether these STACs or other STACs inhibit osteoclast-mediated bone resorption or influence other Sirtuins *in vivo* remains to be investigated in disease models of osteoporosis, aging and impaired metabolism as these conditions are associated with reduced Sirt1 level and function [46,47]. Importantly, our findings may have implications beyond osteoclast biology as they shed novel light on the STACs mechanism of action.

## Supporting Information

S1 Fig The effect of SRT2183 administration on protein content in RANKL-induced BMMs. SRT2183 or vehicle were co-administrated with RANKL. Protein content was determined 4 days post RANKL stimulation. Data are Mean ± SEM (n = 3 independent experiments).
(TIF)

S2 Fig SRT2183 does not influence early MAP Kinase and NFκB phosphorylation. Western blot analysis of phosphorylated and total JNK, Erk1/2, p38 and p65 in SRT2183- and vehicle-treated BMMs 0, 10, 20, 30, 60 minutes post RANKL administration. p- phosphorylated; JNK/Jun-amino-terminal kinase (mitogen-activated protein kinase 8/ mitogen-activated protein kinase 9): Erk1/2, extracellular signal regulated kinase 1/ extracellular signal regulated kinase 2 (mitogen-activated protein kinase 3/ mitogen-activated protein kinase 1); p38 (mitogen-activated protein kinase 14); p65 (RelA, v-rel reticuloendotheliosis viral oncogene homolog A). Data are Mean ± SEM (n = 3 independent experiments).
(TIF)

S3 Fig Sirtuin 2–7 protein expression in RANKL-induced WT and *Sirt1* ^*-/-*^ BMMs. Western blot analysis of Sirt2-7 and GAPDH in vehicle-treated BMMs obtained from WT and *sirt1* knockout (*Sirt1* ^*-/-*^ *)* mice 4 days post RANKL stimulation. Data are Mean ± SEM (n = 3 mice of each genotype).
(TIF)

S4 Fig SRT2183 does not influence Sirt2, 4–7 protein level in WT and in *Sirt1* ^*-/-*^-derived osteoclasts. (A-B) The effect of SRT2183 on protein levels in (A) WT and (B) *Sirt1* ^*-/-*^-derived osteoclasts. Western blot analysis of Sirt2-7, GAPDH and HSP90 in SRT2183- or vehicle-treated osteoclasts 4 days post RANKL stimulation. Data are Mean ± SEM (n = 3 mice of each genotype).
(TIF)

S5 Fig SRT3025 inhibits RANKL-induced osteoclastogenesis in bone marrow-derived macrophages (BMMs). (A) The effects of SRT3025 on osteoclast differentiation. BMMs were inducted to osteoclastogenesis with RANKL in the presence or absence of SRT3025. TRAP staining was performed 4 days post RANKL stimulation. (B) The effect of SRT3025 on pit formation by RANKL-induced osteoclasts. BMMs were inducted to osteoclastogenesis in the presence or absence of SRT3025. A pit formation assay (left panel) and eroded area (right) are shown. (C-D) The effect of SRT3025 on NFATc1 (C) and DC-STAMP (D) expression. Western blot analysis of NFATc1 and GAPDH in SRT3025 or vehicle-treated osteoclasts 4 days post RANKL stimulation. (E) The effect of SRT3025 on expression of osteoclast markers and fusion-related genes. Gene expression analysis by quantitative RT-PCR 4 days post induction to osteoclastogenesis is shown. Results are relative to *GAPDH*. (F) The effect of SRT3025 on AMPK phosphorylation. Western blot analysis of pAMPKα and AMPKα in SRT3025 or vehicle-treated osteoclasts 4 days post RANKL stimulation. (G) The effect of SRT3025 on ACC phosphorylation. Western blot analysis of pACC and ACC in SRT3025 or vehicle-treated osteoclasts 4 days post RANKL stimulation. (H) The effect of SRT3025 on Sirt1 expression in RANKL-stimulated osteoclasts. Western blot analysis of Sirt1and HSP90 in SRT3025 or vehicle-treated osteoclasts 4 days post RANKL stimulation. (I) The effect of SRT3025 on IκBα expression. Western blot analysis of IκBα and GAPDH in SRT3025 or vehicle-treated BMMs 24 hours post RANKL stimulation. (J) The effect of SRT3025 on p65 acetylation (Lys310). Western blot analysis of p65K310 ac and p65 in SRT3025 or vehicle-treated osteoclasts 4 days post RANKL stimulation. Data are Mean ±SEM (n = 3), analyzed by paired sample Student's *t-*test (B) or one-sample Student’s *t*-test (C-J) *P<0.05, **P<0.01, ***P<0.001 compared to vehicle-treated BMMs. Magnification X40; Scale bar 1mm.
(TIF)

S6 Fig SRT3025 inhibits RANKL-induced osteoclastogenesis in *Sirt1* ^*-/-*^ bone marrow-derived macrophages (BMMs). (A) The effect of SRT3025 on osteoclast differentiation. *Sirt1* ^-/-^-derived BMMs were inducted to osteoclastogenesis with RANKL in the presence or absence of SRT3025. TRAP staining was performed 4 days post RANK stimulation. (B) The effect of SRT3025 on pit formation by RANKL-induced BMMs. *Sirt1* ^-/-^-derived BMMs were inducted to osteoclastogenesis in the presence or absence of SRT3025. A pit formation assay (left panel) and eroded area (right) are shown. (C) The effect of SRT3025 on Sirt3 protein level. Western blot analysis of Sirt3 and HSP90 in SRT3025- and vehicle-treated osteoclasts 4 days post RANKL stimulation. Data are Mean ±SEM (n = 3 independent experiments), analyzed by paired Student's *t*-test (B) and one-sample Student's *t*-test (C-E); **P<0.01; ***P<0.001 compared to vehicle-treated BMMs. Magnification X40; Scale bar 1mm.
(TIF)

S1 Text Supplementary Methods. (DOC)

## References

[1] Sebastian C, Satterstrom FK, Haigis MC, Mostoslavsky R. From sirtuin biology to human diseases: an update. J Biol Chem. 2012; 287: 42444–42452. 10.1074/jbc.R112.402768 23086954 PMC3522245

[2] Herranz D, Munoz-Martin M, Canamero M, Mulero F, Martinez-Pastor B, Fernandez-Capetillo O, et al Sirt1 improves healthy ageing and protects from metabolic syndrome-associated cancer. Nat Commun. 2010 4 12;1:3 10.1038/ncomms1001 20975665 PMC3641391

[3] Cohen-Kfir E, Artsi H, Levin A, Abramowitz E, Bajayo A, Gurt I, et al Sirt1 is a regulator of bone mass and a repressor of Sost encoding for sclerostin, a bone formation inhibitor. Endocrinology. 2011 12;152(12):4514–24. 10.1210/en.2011-1128 21952235

[4] Simic P, Zainabadi K, Bell E, Sykes DB, Saez B, Lotinun S, et al SIRT1 regulates differentiation of mesenchymal stem cells by deacetylating beta-catenin. EMBO Mol Med. 2013; 3;5(3):430–40. 10.1002/emmm.201201606 23364955 PMC3598082

[5] Iyer S, Han L, Bartell SM, Kim HN, Gubrij I, de Cabo R, et al Sirtuin1 (Sirt1) promotes cortical bone formation by preventing beta-catenin sequestration by FoxO transcription factors in osteoblast progenitors. J Biol Chem. 2014; 8 29;289(35):24069–78. 10.1074/jbc.M114.561803 25002589 PMC4148840

[6] Edwards JR, Perrien DS, Fleming N, Nyman JS, Ono K, Connelly L, et al Silent information regulator (Sir)T1 inhibits NF-kappaB signaling to maintain normal skeletal remodeling. J Bone Miner Res. 2013; 4;28(4):960–9. 10.1002/jbmr.1824 23172686

[7] Shakibaei M, Buhrmann C, Mobasheri A. Resveratrol-mediated SIRT-1 interactions with p300 modulate receptor activator of NF-kappaB ligand (RANKL) activation of NF-kappaB signaling and inhibit osteoclastogenesis in bone-derived cells. J Biol Chem. 2011; 286: 11492–11505. 10.1074/jbc.M110.198713 21239502 PMC3064204

[8] Nakamura T, Imai Y, Matsumoto T, Sato S, Takeuchi K, Igarashi K, et al Estrogen prevents bone loss via estrogen receptor alpha and induction of Fas ligand in osteoclasts. Cell. 2007; 9 7;130(5):811–23. . 17803905 10.1016/j.cell.2007.07.025

[9] Bowers JL, Tyulmenkov VV, Jernigan SC, Klinge CM. Resveratrol acts as a mixed agonist/antagonist for estrogen receptors alpha and beta. Endocrinology. 2000; 141: 3657–3667. 11014220 10.1210/endo.141.10.7721

[10] Pacholec M, Bleasdale JE, Chrunyk B, Cunningham D, Flynn D, Garofalo RS, et al SRT1720, SRT2183, SRT1460, and resveratrol are not direct activators of SIRT1. J Biol Chem. 2010; 3 12;285(11):8340–51. 10.1074/jbc.M109.088682 20061378 PMC2832984

[11] Borra MT, Smith BC, Denu JM. Mechanism of human SIRT1 activation by resveratrol. J Biol Chem. 2005; 280: 17187–17195. 15749705 10.1074/jbc.M501250200

[12] Kaeberlein M, McDonagh T, Heltweg B, Hixon J, Westman EA, Caldwell SD, et al Substrate-specific activation of sirtuins by resveratrol. J Biol Chem. 2005 4 29;280(17):17038–45. . 15684413 10.1074/jbc.M500655200

[13] Beher D, Wu J, Cumine S, Kim KW, Lu SC, Atangan L, et al Resveratrol is not a direct activator of SIRT1 enzyme activity. Chem Biol Drug Des. 2009 12;74(6):619–24. 10.1111/j.1747-0285.2009.00901.x E 19843076

[14] Hubbard BP, Gomes AP, Dai H, Li J, Case AW, Considine T, et al Evidence for a common mechanism of SIRT1 regulation by allosteric activators. Science. 2013 3 8;339(6124):1216–9. 10.1126/science.1231097 23471411 PMC3799917

[15] National Institute of Mental Health; University of Virginia A Study in Healthy Male Volunteers to Investigate Different Doses of a New Drug for the Treatment of Metabolic Diseases In: ClinicalTrials.gov [Internet]. Bethesda (MD): National Library of Medicine (US) 2011–2012 Available from: http://clinicaltrials.gov/show/NCT01340911. NLM Identifier: NCT01340911

[16] National Institute of Mental Health; University of Virginia. A Phase 1b Study to Assess the Safety and Anti-inflammatory Effects of Two Different Doses of SRT2104 in Patients With Ulcerative Colitis In: ClinicalTrials.gov [Internet]. Bethesda (MD): National Library of Medicine (US) 2011–2013 Available from: http://clinicaltrials.gov/show/NCT01453491 NLM Identifier: NCT01453491.

[17] National Institute of Mental Health; University of Virginia. A Clinical Study to Assess the Safety, Tolerability, and Activity of Oral SRT2104 Capsules Administered for 28 Days to Subjects With Type 2 Diabetes Mellitus In: ClinicalTrials.gov [Internet]. Bethesda (MD): National Library of Medicine (US) 2009–2012 Available from: http://clinicaltrials.gov/show/NCT01018017. NLM Identifier: NCT01018017.

[18] Indo Y, Takeshita S, Ishii KA, Hoshii T, Aburatani H, Hirao A, et al Metabolic regulation of osteoclast differentiation and function. J Bone Miner Res. 2013 11;28(11):2392–9. 10.1002/jbmr.1976 23661628

[19] Hubbard BP, Sinclair DA. Small molecule SIRT1 activators for the treatment of aging and age-related diseases. Trends Pharmacol Sci. 2014; 35: 146–154. 10.1016/j.tips.2013.12.004 24439680 PMC3970218

[20] Cheng HL, Mostoslavsky R, Saito S, Manis JP, Gu Y, Patel P, et al Developmental defects and p53 hyperacetylation in Sir2 homolog (SIRT1)-deficient mice. Proc Natl Acad Sci U S A. 2003 9 16;100(19):10794–9. 12960381 10.1073/pnas.1934713100 PMC196882

[21] Takeshita S, Kaji K, Kudo A. Identification and characterization of the new osteoclast progenitor with macrophage phenotypes being able to differentiate into mature osteoclasts. J Bone Miner Res. 2000; 15: 1477–1488. 10934646 10.1359/jbmr.2000.15.8.1477

[22] Kartner N, Yao Y, Li K, Crasto GJ, Datti A, Manolson MF. Inhibition of osteoclast bone resorption by disrupting vacuolar H+-ATPase a3-B2 subunit interaction. J Biol Chem. 2010 11 26;285(48):37476–90. 10.1074/jbc.M110.123281 20837476 PMC2988353

[23] Takayanagi H, Kim S, Koga T, Nishina H, Isshiki M, Yoshida H, et al Induction and activation of the transcription factor NFATc1 (NFAT2) integrate RANKL signaling in terminal differentiation of osteoclasts. Dev Cell. 2002 12;3(6):889–901. 12479813 10.1016/s1534-5807(02)00369-6

[24] Yagi M, Miyamoto T, Sawatani Y, Iwamoto K, Hosogane N, Fujita N, et al DC-STAMP is essential for cell-cell fusion in osteoclasts and foreign body giant cells. J Exp Med. 2005 8 1;202(3):345–51. . 16061724 10.1084/jem.20050645 PMC2213087

[25] Ruderman NB, Xu XJ, Nelson L, Cacicedo JM, Saha AK, Lan F et al AMPK and SIRT1: a long-standing partnership? Am J Physiol Endocrinol Metab. 2010 4;298(4):E751–60. 10.1152/ajpendo.00745.2009 20103737 PMC2853213

[26] Kang H, Viollet B, Wu D. Genetic deletion of catalytic subunits of AMP-activated protein kinase increases osteoclasts and reduces bone mass in young adult mice. J Biol Chem. 2013; 288: 12187–12196. 10.1074/jbc.M112.430389 23486478 PMC3636902

[27] Canto C, Gerhart-Hines Z, Feige JN, Lagouge M, Noriega L, Milne JC, et al AMPK regulates energy expenditure by modulating NAD+ metabolism and SIRT1 activity. Nature. 2009 4 23;458(7241):1056–60. 10.1038/nature07813 19262508 PMC3616311

[28] Novack DV. Role of NF-kappaB in the skeleton. Cell Res. 2011; 21: 169–182. 10.1038/cr.2010.159 21079651 PMC3193402

[29] Yeung F, Hoberg JE, Ramsey CS, Keller MD, Jones DR, Frye RA, et al Modulation of NF-kappaB-dependent transcription and cell survival by the SIRT1 deacetylase. Embo J. 2004 6 16;23(12):2369–80. . 15152190 10.1038/sj.emboj.7600244 PMC423286

[30] Hattori Y, Suzuki K, Hattori S, Kasai K. Metformin inhibits cytokine-induced nuclear factor kappaB activation via AMP-activated protein kinase activation in vascular endothelial cells. Hypertension. 2006; 47: 1183–1188. 16636195 10.1161/01.HYP.0000221429.94591.72

[31] Qiu X, Brown K, Hirschey MD, Verdin E, Chen D. Calorie restriction reduces oxidative stress by SIRT3-mediated SOD2 activation. Cell Metab. 2010; 12: 662–667. 10.1016/j.cmet.2010.11.015 21109198

[32] Chen LF, Mu Y, Greene WC. Acetylation of RelA at discrete sites regulates distinct nuclear functions of NF-kappaB. Embo J. 2002; 21: 6539–6548. 12456660 10.1093/emboj/cdf660 PMC136963

[33] Vaira S, Alhawagri M, Anwisye I, Kitaura H, Faccio R, Novack DV. RelA/p65 promotes osteoclast differentiation by blocking a RANKL-induced apoptotic JNK pathway in mice. J Clin Invest. 2008 6;118(6):2088–97. 10.1172/JCI33392 18464930 PMC2373419

[34] Chen L, Fischle W, Verdin E, Greene WC. Duration of nuclear NF-kappaB action regulated by reversible acetylation. Science. 2001; 293: 1653–1657. 11533489 10.1126/science.1062374

[35] Yang H, Zhang W, Pan H, Feldser HG, Lainez E, Miller C, et al SIRT1 activators suppress inflammatory responses through promotion of p65 deacetylation and inhibition of NF-kappaB activity. PLoS One. 2012;7(9):e46364 10.1371/journal.pone.0046364 23029496 PMC3460821

[36] Lan F, Cacicedo JM, Ruderman N, Ido Y. SIRT1 modulation of the acetylation status, cytosolic localization, and activity of LKB1. Possible role in AMP-activated protein kinase activation. J Biol Chem. 2008; 283: 27628–27635. 10.1074/jbc.M805711200 18687677 PMC2562073

[37] Fulco M, Cen Y, Zhao P, Hoffman EP, McBurney MW, Sauve AA, et al Glucose restriction inhibits skeletal myoblast differentiation by activating SIRT1 through AMPK-mediated regulation of Nampt. Dev Cell. 2008 5;14(5):661–73. 10.1016/j.devcel.2008.02.004 18477450 PMC2431467

[38] Bess E, Fisslthaler B, Fromel T, Fleming I. Nitric oxide-induced activation of the AMP-activated protein kinase alpha2 subunit attenuates IkappaB kinase activity and inflammatory responses in endothelial cells. PLoS One. 2011; 6: e20848 10.1371/journal.pone.0020848 21673972 PMC3108981

[39] Mercken EM, Mitchell SJ, Martin-Montalvo A, Minor RK, Almeida M, Gomes AP, et al SRT2104 extends survival of male mice on a standard diet and preserves bone and muscle mass. Aging Cell. 2014 10;13(5):787–96. 10.1111/acel.12220 24931715 PMC4172519

[40] Huber JL, McBurney MW, Distefano PS, McDonagh T. SIRT1-independent mechanisms of the putative sirtuin enzyme activators SRT1720 and SRT2183. Future Med Chem. 2010; 2: 1751–1759. 10.4155/fmc.10.257 21428798

[41] Nguyen GT, Schaefer S, Gertz M, Weyand M, Steegborn C. Structures of human sirtuin 3 complexes with ADP-ribose and with carba-NAD+ and SRT1720: binding details and inhibition mechanism. Acta Crystallogr D Biol Crystallogr. 2013; 69: 1423–1432. 10.1107/S0907444913015448 23897466

[42] Jin L, Galonek H, Israelian K, Choy W, Morrison M, Xia Y, et al Biochemical characterization, localization, and tissue distribution of the longer form of mouse SIRT3. Protein Sci. 2009 3;18(3):514–25. 10.1002/pro.50 19241369 PMC2760358

[43] Gertz M, Nguyen GT, Fischer F, Suenkel B, Schlicker C, Fränzel B, et al A molecular mechanism for direct sirtuin activation by resveratrol. PLoS One. 2012;7(11):e49761 10.1371/journal.pone.0049761 23185430 PMC3504108

[44] Deng FY, Lei SF, Chen XD, Tan LJ, Zhu XZ, Deng HW. An integrative study ascertained SOD2 as a susceptibility gene for osteoporosis in Chinese. J Bone Miner Res. 2011 11;26(11):2695–701. 10.1002/jbmr.471 21773993 PMC3375319

[45] Deng FY, Liu YZ, Li LM, Jiang C, Wu S, Chen Y, et al Proteomic analysis of circulating monocytes in Chinese premenopausal females with extremely discordant bone mineral density. Proteomics. 2008 10;8(20):4259–72. 10.1002/pmic.200700480 18924182 PMC2760933

[46] Chalkiadaki A, Guarente L. High-fat diet triggers inflammation-induced cleavage of SIRT1 in adipose tissue to promote metabolic dysfunction. Cell Metab. 2012; 16: 180–188. 10.1016/j.cmet.2012.07.003 22883230 PMC3539750

[47] Han L, Zhou R, Niu J, McNutt MA, Wang P, Tong T. SIRT1 is regulated by a PPAR{gamma}-SIRT1 negative feedback loop associated with senescence. Nucleic Acids Res. 2010 11;38(21):7458–71. 10.1093/nar/gkq609 20660480 PMC2995042

