# Supplementary figures and images for: The Sirt1 Activators SRT2183 and SRT3025 Inhibit RANKL-Induced Osteoclastogenesis in Bone Marrow-Derived Macrophages and Down-Regulate Sirt3 in Sirt1 Null Cells
Source: PLoS One. 2015 Jul 30;10(7):e0134391. doi: 10.1371/journal.pone.0134391 (PMC4520518; doi:10.1371/journal.pone.0134391)

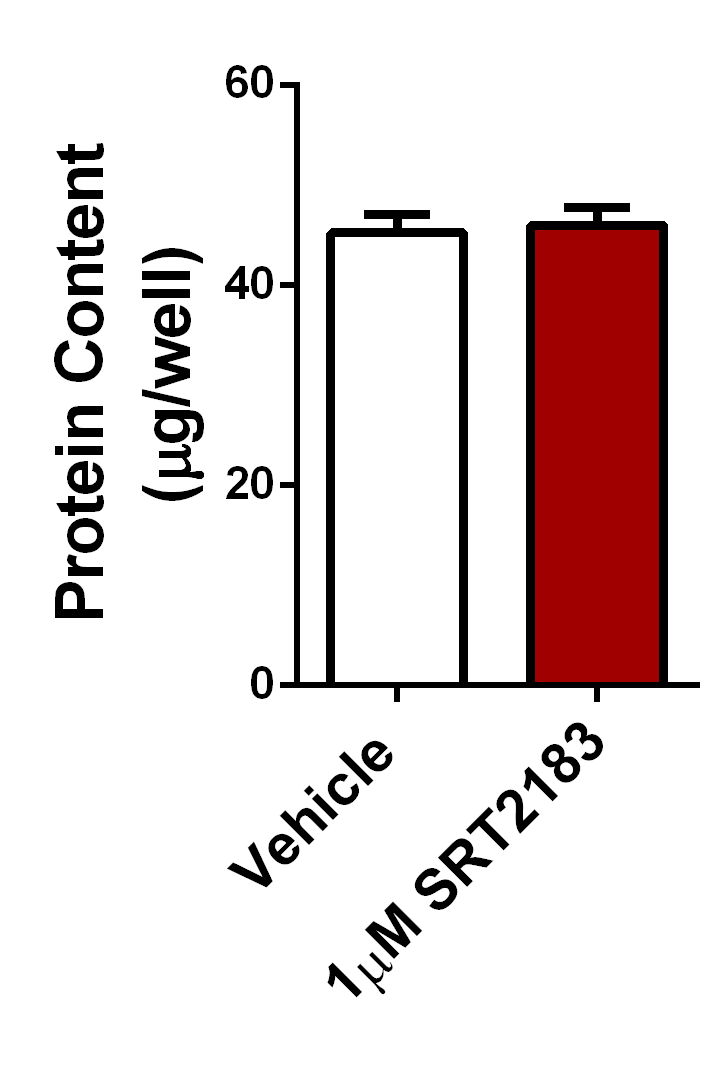

Supplement: S1 Fig — SRT2183 or vehicle were co-administrated with RANKL. Protein content was determined 4 days post RANKL stimulation. Data are Mean ± SEM (n = 3 independent experiments). (TIF) [file pone.0134391.s001.tif]

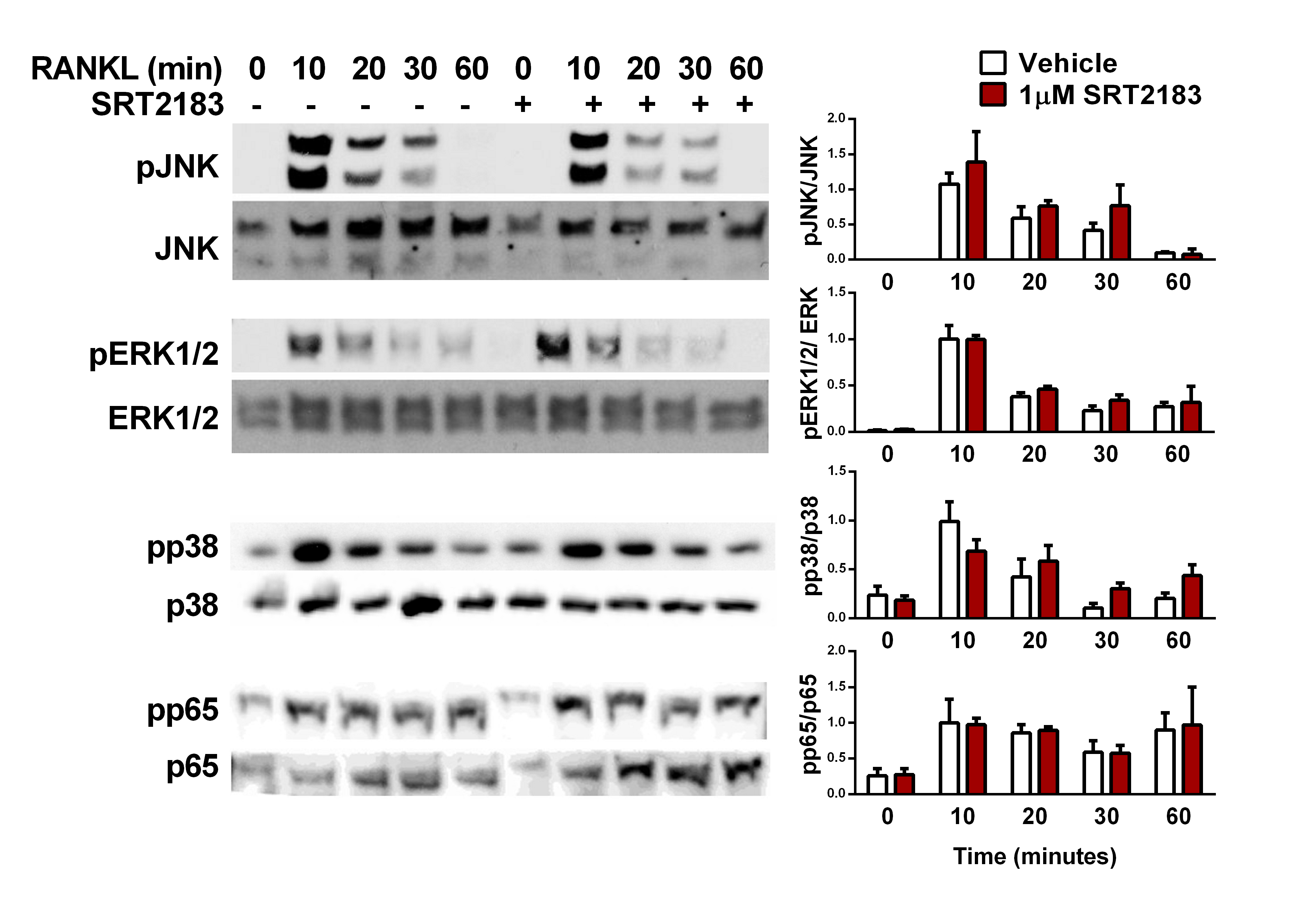

Supplement: S2 Fig — Western blot analysis of phosphorylated and total JNK, Erk1/2, p38 and p65 in SRT2183- and vehicle-treated BMMs 0, 10, 20, 30, 60 minutes post RANKL administration. p- phosphorylated; JNK/Jun-amino-terminal kinase (mitogen-activated protein kinase 8/ mitogen-activated protein kinase 9): Erk1/2, extracellular signal regulated kinase 1/ extracellular signal regulated kinase 2 (mitogen-activated protein kinase 3/ mitogen-activated protein kinase 1); p38 (mitogen-activated protein kinase 14); p65 (RelA, v-rel reticuloendotheliosis viral oncogene homolog A). Data are Mean ± SEM (n = 3 independent experiments). (TIF) [file pone.0134391.s002.tif]

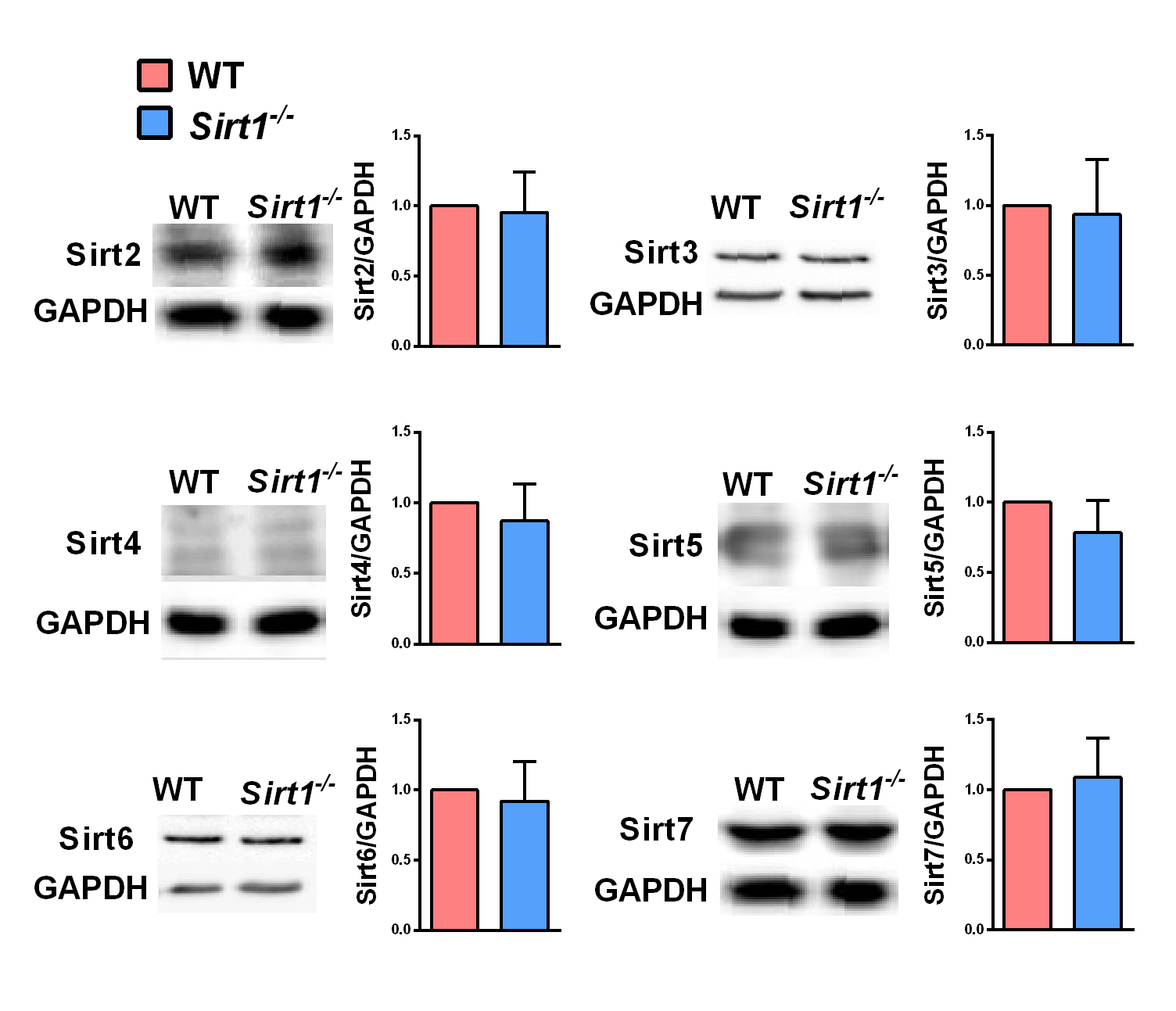

Supplement: S3 Fig — Western blot analysis of Sirt2-7 and GAPDH in vehicle-treated BMMs obtained from WT and sirt1 knockout (Sirt1 -/- ) mice 4 days post RANKL stimulation. Data are Mean ± SEM (n = 3 mice of each genotype). (TIF) [file pone.0134391.s003.tif]

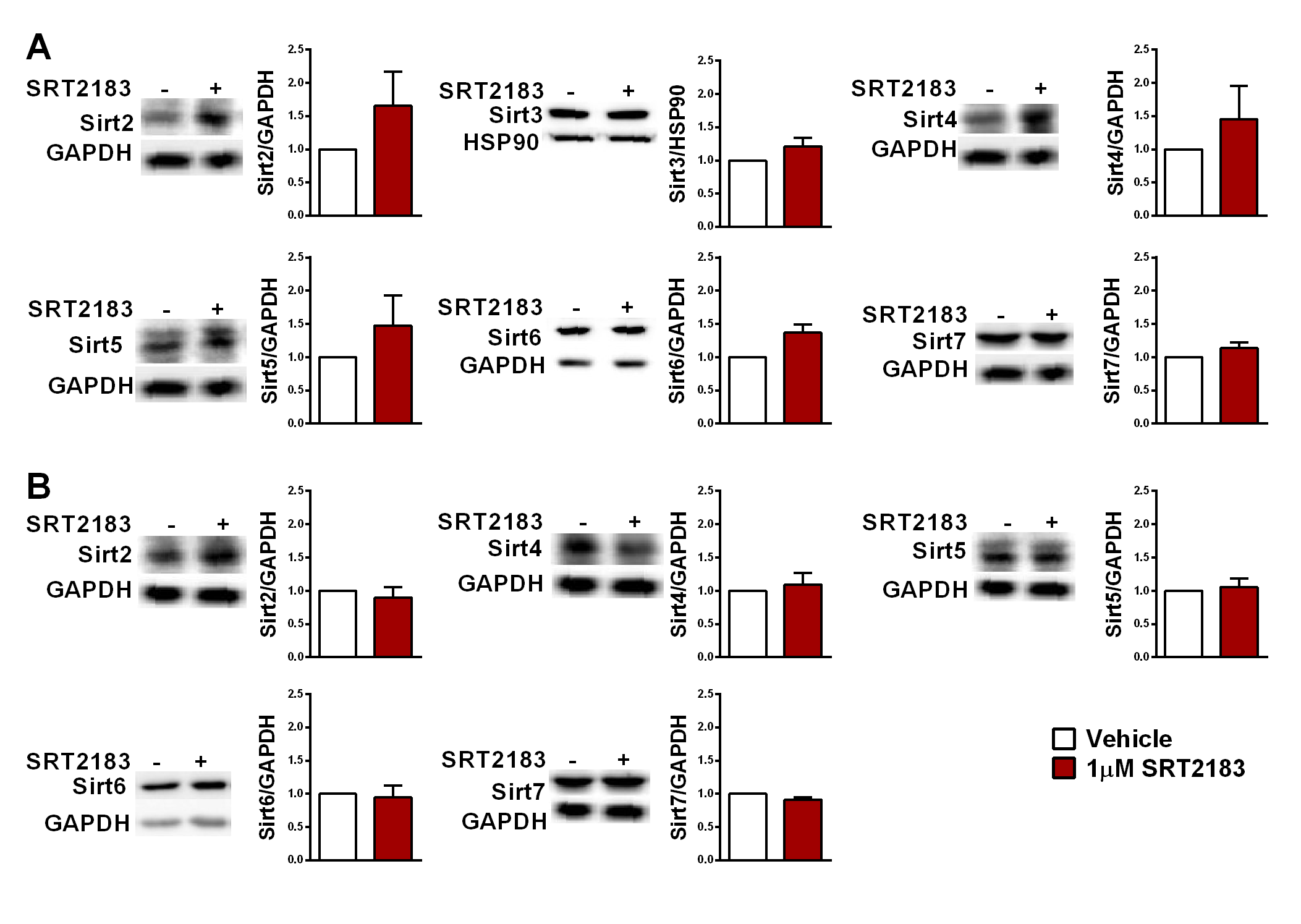

Supplement: S4 Fig — (A-B) The effect of SRT2183 on protein levels in (A) WT and (B) Sirt1 -/--derived osteoclasts. Western blot analysis of Sirt2-7, GAPDH and HSP90 in SRT2183- or vehicle-treated osteoclasts 4 days post RANKL stimulation. Data are Mean ± SEM (n = 3 mice of each genotype). (TIF) [file pone.0134391.s004.tif]

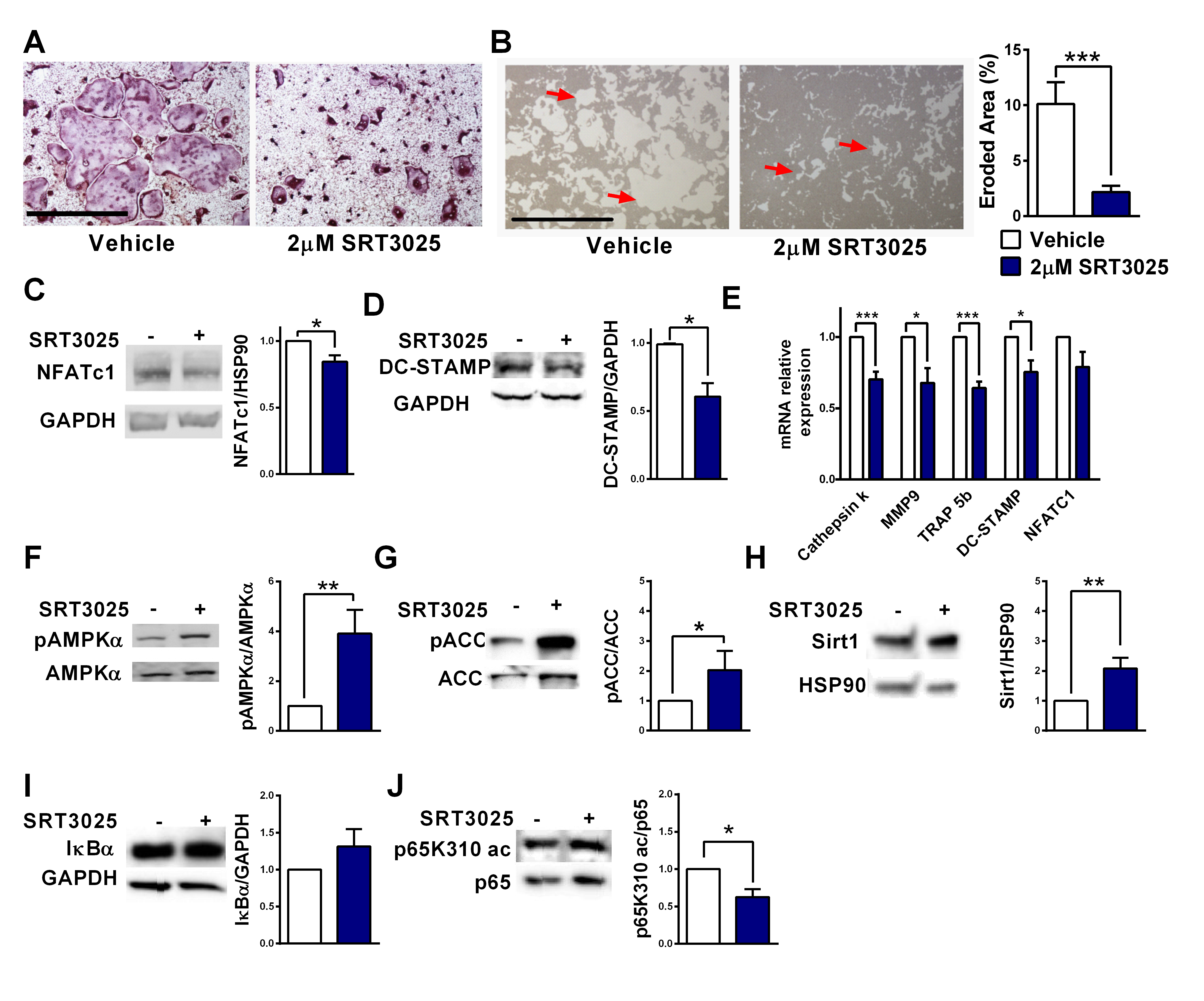

Supplement: S5 Fig — (A) The effects of SRT3025 on osteoclast differentiation. BMMs were inducted to osteoclastogenesis with RANKL in the presence or absence of SRT3025. TRAP staining was performed 4 days post RANKL stimulation. (B) The effect of SRT3025 on pit formation by RANKL-induced osteoclasts. BMMs were inducted to osteoclastogenesis in the presence or absence of SRT3025. A pit formation assay (left panel) and eroded area (right) are shown. (C-D) The effect of SRT3025 on NFATc1 (C) and DC-STAMP (D) expression. Western blot analysis of NFATc1 and GAPDH in SRT3025 or vehicle-treated osteoclasts 4 days post RANKL stimulation. (E) The effect of SRT3025 on expression of osteoclast markers and fusion-related genes. Gene expression analysis by quantitative RT-PCR 4 days post induction to osteoclastogenesis is shown. Results are relative to GAPDH. (F) The effect of SRT3025 on AMPK phosphorylation. Western blot analysis of pAMPKα and AMPKα in SRT3025 or vehicle-treated osteoclasts 4 days post RANKL stimulation. (G) The effect of SRT3025 on ACC phosphorylation. Western blot analysis of pACC and ACC in SRT3025 or vehicle-treated osteoclasts 4 days post RANKL stimulation. (H) The effect of SRT3025 on Sirt1 expression in RANKL-stimulated osteoclasts. Western blot analysis of Sirt1and HSP90 in SRT3025 or vehicle-treated osteoclasts 4 days post RANKL stimulation. (I) The effect of SRT3025 on IκBα expression. Western blot analysis of IκBα and GAPDH in SRT3025 or vehicle-treated BMMs 24 hours post RANKL stimulation. (J) The effect of SRT3025 on p65 acetylation (Lys310). Western blot analysis of p65K310 ac and p65 in SRT3025 or vehicle-treated osteoclasts 4 days post RANKL stimulation. Data are Mean ±SEM (n = 3), analyzed by paired sample Student's t-test (B) or one-sample Student’s t-test (C-J) *P<0.05, **P<0.01, ***P<0.001 compared to vehicle-treated BMMs. Magnification X40; Scale bar 1mm. (TIF) [file pone.0134391.s005.tif]

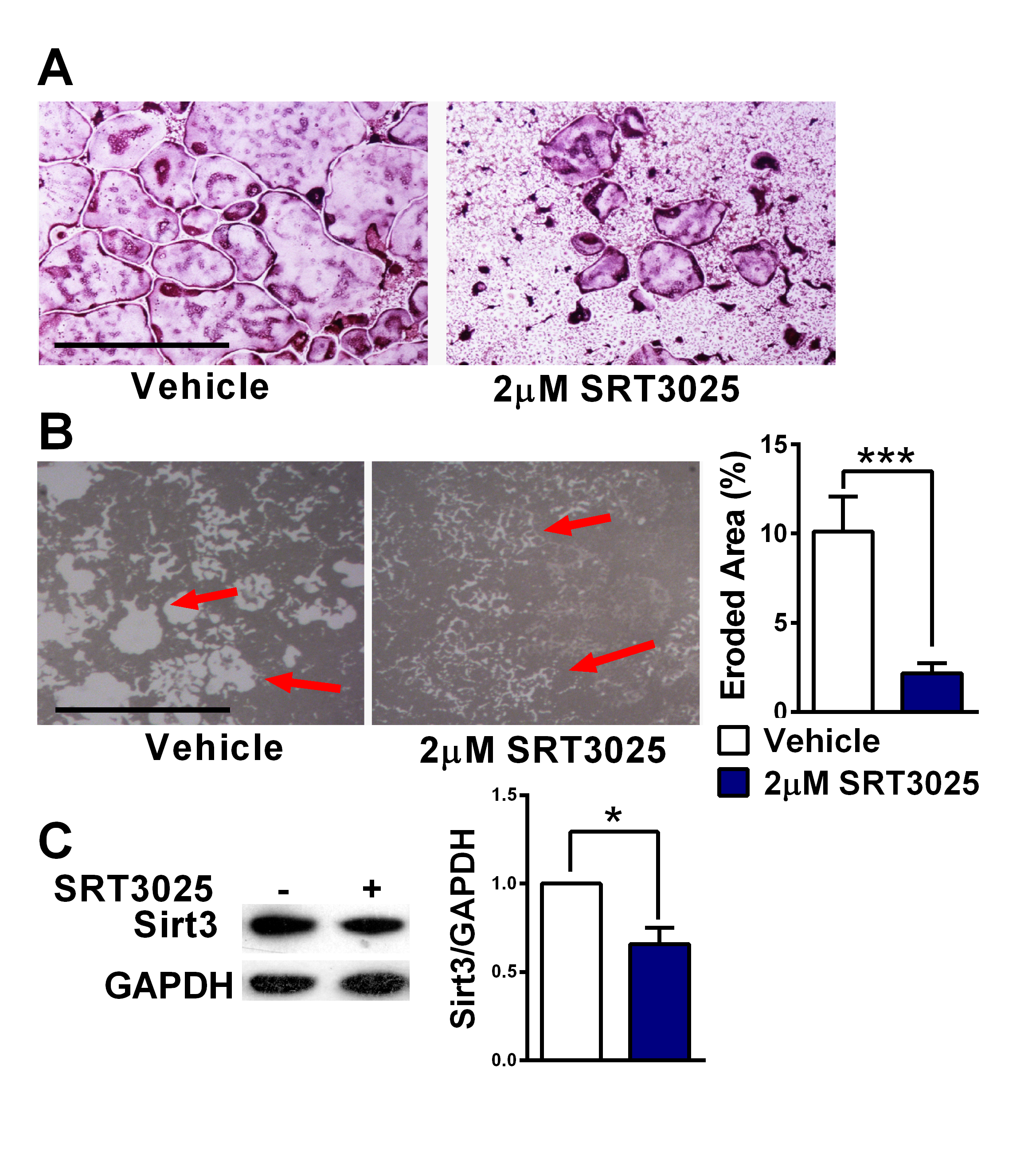

Supplement: S6 Fig — (A) The effect of SRT3025 on osteoclast differentiation. Sirt1 -/--derived BMMs were inducted to osteoclastogenesis with RANKL in the presence or absence of SRT3025. TRAP staining was performed 4 days post RANK stimulation. (B) The effect of SRT3025 on pit formation by RANKL-induced BMMs. Sirt1 -/--derived BMMs were inducted to osteoclastogenesis in the presence or absence of SRT3025. A pit formation assay (left panel) and eroded area (right) are shown. (C) The effect of SRT3025 on Sirt3 protein level. Western blot analysis of Sirt3 and HSP90 in SRT3025- and vehicle-treated osteoclasts 4 days post RANKL stimulation. Data are Mean ±SEM (n = 3 independent experiments), analyzed by paired Student's t-test (B) and one-sample Student's t-test (C-E); **P<0.01; ***P<0.001 compared to vehicle-treated BMMs. Magnification X40; Scale bar 1mm. (TIF) [file pone.0134391.s006.tif]
