# Supplementary material for: The Sirt1 Activators SRT2183 and SRT3025 Inhibit RANKL-Induced Osteoclastogenesis in Bone Marrow-Derived Macrophages and Down-Regulate Sirt3 in Sirt1 Null Cells
Source: PLoS One. 2015 Jul 30;10(7):e0134391. doi: 10.1371/journal.pone.0134391 (PMC4520518; doi:10.1371/journal.pone.0134391)
Supplement: S1 Text — (DOC) [file pone.0134391.s007.doc]

**S7**

**Methods:**

***Protein content***

Bone marrow-derived macrophages (BMMs) from femurs and tibias were collected, plated, and non-adherent cells were re-plated 24-hrs later in a 96-well plate at a concentration of 20,000 cells/well. The cells were cultured for 3 days in 5% CMG14–12 culture supernatant as a source of macrophage-colony stimulating factor (M-CSF) in minimum essential medium α (α–MEM) containing 15% FBS. The plated cells were then induced to differentiation with 10% M-CSF and 10 ng/ml RANKL (PeproTech, Rocky Hill, New Jersey) for 4 days with a medium change every 3 days. 1µM SRT2183 or a vehicle was co-administrated with RANKL. 4 days post induction to osteoclastogenesis cells were collected in RIPA buffer and total protein was measure with Protein Assay Dye Reagent Concentrate (cat.no.500-0006, Bio-Rad Laboratories, GmbH).

***Signaling pathways studies:***

For the signaling pathway studies cells were cultured in α–MEM/15%FBS/5% M-CSF for 3 days and then in serum free medium for 12 hours. SRT2183 or a vehicle was added 1 hour prior to RANKL (15ng/ml) stimulation, and cell lysates were harvested at the indicated times (0, 10, 20, 30, 60 minutes).

The following antibodies were used for the signaling pathways studies: phosphorylated stress-activated protein kinase/Jun-amino-terminal kinase (mitogen-activated protein kinase 8/mitogen-activated protein kinase 9, phospho-SAPK/JNK; #4668, Cell Signaling), SAPK/JNK (#9252, Cell Signaling), phosphorylated extracellular signal regulated kinase (mitogen-activated protein kinase 1/mitogen-activated protein kinase 3, phospho-ERK1/2; #4376, Cell Signaling), ERK1/2 (#4695, Cell Signaling); phosphorylated p38 (mitogen-activated protein kinase 14; sc-17852, Santa Cruz), p38α (sc-535, Santa Cruz), phosphorylated NF-κB p65 (Rela, v-rel reticuloendotheliosis viral oncogene homolog A; #3033, Cell Signaling).

***Sirtuin2, 4-7 protein expression determination:***

The following antibodies were used for Sirtuins protein level determination by western blot analysis: Sirt2 (sc-28298, Santa Cruz), Sirt4 (sc-135053, Santa Cruz), Sirt5 (sc-66273, Santa Cruz), Sirt2 (sc-28298, Santa Cruz), Sirt6 (ab62739, Abcam), Sirt7 (sc-135055, Santa Cruz).
